# The Relationship Between Mitral Annular Calcification, Metabolic Syndrome and Thromboembolic Risk

**DOI:** 10.21470/1678-9741-2019-0062

**Published:** 2019

**Authors:** Fatih Aksoy, Serdar Guler, Fatih Kahraman, Mevlüt Serdar Kuyumcu, Ali Bagcı, Hasan Aydın Bas, Dinçer Uysal, Ercan Varol

**Affiliations:** 1 Department of Cardiology, Suleyman Demirel University, Medical School, Isparta, Turkey.; 2 Department of Cardiovascular Surgery, Suleyman Demirel University, Medical School, Isparta, Turkey.

**Keywords:** Atrial Fibrilation, Stroke, Metabolic Syndrome, Coronary Artery Disease, Brain Ischemia, Thromboembolism, Hypertension

## Abstract

**Introduction:**

Metabolic syndrome (MetS) is defined as an association between diabetes, hypertension, obesity and dyslipidemia and an increased risk of cardiovascular disease. Mitral annular calcification (MAC) is associated with several cardiovascular disorders, including coronary artery disease, atrial fibrillation (AF), heart failure, ischemic stroke and increased mortality. The CHA_2_DS_2_-VASc score is used to estimate thromboembolic risk in AF. However, the association among MAC, MetS and thromboembolic risk is unknown and was evaluated in the current study.

**Methods:**

The study group consisted of 94 patients with MAC and 86 patients with MetS. Patients were divided into two groups: those with and those without MAC.

**Results:**

Patients with MAC had a higher MetS rate (*P*<0.001). In patients with MAC, the CHA_2_DS_2_-VASc scores and the rate of cerebrovascular accident and AF were significantly higher compared to those without MAC (*P*<0.001, for both parameters). The results of the multivariate regression analysis showed that history of smoking, presence of MetS and high CHA_2_DS_2_-VASc scores were associated with the development of MAC. ROC curve analyses showed that CHA_2_DS_2_-VASc scores were signiﬁcant predictors for MAC (C-statistic: 0.78; 95% CI: 0.706-0.855, *P*<0.001). Correlation analysis indicated that MAC was positively correlated with the presence of MetS and CHA_2_DS_2_-VASc score (*P*=0.001, r=0.264; *P*<0.001, r=0.490).

**Conclusion:**

We have shown that CHA_2_DS_2_-VASc score and presence of MetS rates were significantly higher in patients with MAC compared without MAC. Presence of MAC was correlated with CHA_2_DS_2_-VASc score, presence of MetS, AF and left atrial diameter and negatively correlated with left ventricular ejection fraction.

**Table:** 

Abbreviations, acronyms & symbols				
AF	= Atrial fibrillation		LVESD	= Left ventricular end-systolic diameter
BMP	= Bone morphogenic protein		LVPW	= Left ventricular posterior wall
CI	= Confidence interval		MAC	= Mitral annular calcification
CVA	= Cerebrovascular accident		MetS	= Metabolic syndrome
HDL	= High-density lipoprotein		MESA	= Multi-Ethnic Study of Atherosclerosis
IQR	= Interquartile range		OR	= Odds ratio
IVS	= Interventricular septum		OS	= Oxidative stress
LA	= Left atrial		ROC	= Receiver operating characteristics
LVEF	= Left ventricular ejection fraction		TIA	= Transient ischemic attack
LVEDD	= Left ventricular end-diastolic diameter			

## INTRODUCTION

Mitral annular calcification (MAC) is a chronic, age-related, non-inflammatory degenerative process affecting the fibrous annulus of the mitral valve^[1-3]^. Recent studies demonstrated the strong association between the coexistence of MAC and cardiovascular risk factors^[1,2]^. In addition, studies have shown a relationship between MAC and carotid and peripheral artery disease^[1,4]^. MAC is accompanied by mitral valve disease and arrhythmias, including atrial fibrillation (AF) and conduction system disease and influenced the outcome of cardiac surgery^[5,6]^. Although it is most commonly asymptomatic and an incidental finding, its clinical relevance comes from the association of MAC with an increased rate of mortality and cardiovascular disease^[7]^. Additionally, a strong correlation was shown between MAC and risk of stroke^[8]^. Coexistence of MAC and stroke can be explained by cardiovascular risk factors, mitral valve disease and arrhythmias, including AF.

Metabolic syndrome (MetS) is a cluster of conditions including metabolic abnormalities, abdominal obesity, high blood pressure, hyperglycemia, low high-density lipoprotein (HDL) cholesterol levels, and high triglyceride levels. Additionally, patients with MetS have higher risk for heart disease, stroke and diabetes^[9]^.

The CHA_2_DS_2_-VASc risk score is an inexpensive and easy scoring system that is calculated by assigning a score of 1 point for each of the following conditions: congestive heart failure (ejection fraction <40%), hypertension, age between 65 and 74 years, diabetes mellitus, vascular disease (myocardial infarction or peripheral arterial disease) and female gender; a score of 2 points for the following conditions: history of stroke or transient ischemic attack (TIA) and age >75 years. The CHA_2_DS_2_-VASc risk score is used to predict the risk of thromboembolism in patients with non-valvular AF^[10]^.

In this study, we aimed to investigate the relationship between MAC, MetS and thromboembolic risk.

## METHODS

### Study Population

The 164 consecutive patients (78 females, 86 males, mean age 70.6±6.3 years) admitted to Suleyman Demirel University Hospital, Department of Cardiology outpatient clinic, and referred to our echocardiography laboratory due to suspicion of heart disease between June 2015 and May 2016 were enrolled in this prospective study. All patients underwent medical history, physical examination, anthropometric measurements, electrocardiogram, and echocardiographic evaluation. The study was approved by the institutional ethics committee and all patients gave their informed consent. Exclusion criteria were pericardial effusion, poor echocardiographic window, and history of chronic renal and liver disease, moderate to severe mitral and aortic regurgitation, moderate to severe mitral and aortic stenosis, malignancy, systemic or pulmonary embolism, chronic hematological diseases, acute or chronic inflammatory disease, autoimmune disease, hyperparathyroidism, hypercalcemia, hyperphosphatemia and presence of prosthetic valve. Patients were divided into two groups, according to the presence of MAC in echocardiographic evaluation.

### Echocardiography

The M-mode, 2D and Doppler echocardiographic examinations were obtained by GE VingMed System FiVe (Norway) to assess left atrial (LA) diameter, interventricular septum (IVS) thickness, left ventricular posterior wall (LVPW) thickness, left ventricular-end diastolic diameter (LVEDD), left ventricular end-systolic diameter (LVESD), left ventricular ejection fraction (LVEF) and MAC. Left atrial and ventricular dimensions and LVEF were measured by M-mode echocardiography in the parasternal long axis view using the American Echocardiography Society M-mode technique^[11]^. MAC was defined as an intense echocardiographic-producing structure with highly reflective characteristics that was located at the junction of the atrioventricular groove and the posterior or anterior mitral leaflet on the parasternal long-axis, apical 4-chamber or 2-chamber, or parasternal short-axis view^[12]^. The presence of mitral and aortic insufficiency was evaluated by Doppler color flow mapping.

### Metabolic Syndrome

At inclusion, MetS was defined according to the National Cholesterol Education Program Adult Treatment Panel III (NCEP ATP III) criteria as ≥3 of the following: (i) waist circumference ≥88 cm in women and ≥102 cm in men; (ii) elevated plasma triglycerides (≥1.7 mmol/L), or treatment for high triglycerides; (iii) low-plasma HDL-C (<1.3 mmol/L for women and <1.04 mmol/L for men); (iv) high fasting plasma glucose (≥5.6 mmol/L), or diabetes treatment; and (v) blood pressure ≥130/80 mmHg, or treated hypertension^[13]^.

### Statistical Analysis

SPSS version 16.0 software package was used for statistical analyses in this study. Categorical variables were expressed as frequency (%) and compared with the χ2 test. Kolmogorov-Smirnov test was used to test the distribution of numerical variables; those with normal distribution were expressed as mean ± standard deviation and were compared with Student’s t-test. Data without normal distribution were expressed as median [interquartile range (IQR) of 25%-75% percentiles] and were compared with the Mann-Whitney U test. In all statistical analyses, *P*-value <0.05 was considered statistically significant. The correlations between CHA_2_DS_2_-VASc risk score, presence of MetS, presence of MAC and other clinical, laboratory and echocardiographic parameters were performed with Pearson or Spearman correlation analysis when appropriate. Univariate analysis of binary logistic regression was carried out to identify which factors were associated with the presence of MAC. After including each of these potential confounding factors, backward conditional binary logistic regression analysis was performed to estimate the odds ratio (OR) and 95% confidence interval (95% CI) for presence of MAC. Receiver operating characteristics (ROC) curve analysis was used to analyze the prognostic value of CHA_2_DS_2_-VASc score for presence of MAC. C-Statistic (area under the curve) was presented as a unified estimate of sensitivity and specificity.

## RESULTS

A total of 164 patients (mean age: 70.6 ± 6.3 years; range 52-85 years) were included in this study. In the echocardiographic evaluation, MAC was diagnosed in 94 patients. Demographic, clinical, laboratory and echocardiographic characteristics of the patients with and without MAC are listed in Table 1. Patients with MAC were generally older, more diabetic and more hypertensive when compared to patients without MAC (*P*<0.001, *P*<0.001 and *P*=0.001, respectively).

**Table 1:** Demographic, clinical, laboratory and echocardiographic characteristics of the patients with and without MAC.

	MAC (-) (n=70)	MAC (+) (n=94)	*P*-value
Age, years	68.4±4.5	72.2±7.0	<0.001
Female gender, n (%)	30 (42)	45 (51)	0.189
Hypertension, n (%)	29 (41)	66 (70)	<0.001
Diabetes mellitus, n (%)	17 (24)	47 (50)	0.001
Hyperlipidemia, n (%)	33 (47)	45 (47)	0.526
Smoking, n (%)	19 (27)	42 (44)	0.016
CAD, n (%)	8 (11)	23 (24.5)	0.026
CVA/TIA, n (%)	1 (1.4)	12 (12.8)	0.006
BMI (kg/m^2^)	29.0±5.1	30.9±7.2	0.06
Waist circumference (cm)	97.4±12.4	101.0±11.0	0.05
SBP (mmHg)	118.0±17	121.0±17	0.182
DBP (mmHg)	75.0±8.1	77.0±10.3	0.09
Heart rate (beat/min)	70.1±12.4	71.7± 13.6	0.41
Statin, n (%)	16 (22)	25 (26)	0.359
ACEi, n (%)	13 (18)	25 (26)	0.154
ARB, n (%)	14 (20)	24 (25)	0.261
OAD, n (%)	7 (10)	32 (34)	<0.001
Insulin, n (%)	4 (5.7)	11 (11.7)	0.149
MetS, n (%)	26 (37)	60 (63)	0.001
CHA_2_DS_2_-VASc score	2.1±1.3	3.5±1.3	<0.001
High risk group for CHA_2_DS_2_-VASc score	42 (60)	86 (91)	<0.001
Platelet count (× 10^3^/µL)	248±81	242±61	0.58
White blood cell count (× 10^3^/µL)	8.032±2.137	7.775±2.618	0.50
HDL-cholesterol, mg/dL	47.1±15.9	47.1±11.9	0.99
LDL-cholesterol, mg/dL	108.4±38.8	113.6±38.3	0.40
Triglycerides, mg/dL	154.1±119.22	147.9±75.3	0.70
Glucose, mg/dL	122.6±58.6	123.9±57.6	0.88
Creatinine, mg/dL)	0.9 ±0.1	0.9 ± 0.1	0.62
Total cholesterol, mg/dL	184.7±57.5	190±47.4	0.47
LVEF, %	60 ±2.2	58.7±4.1	0.02
Aorta (mm)	24.9 ±2.3	25.9±2.3	0.005
LA (mm)	34.7±4.8	37.4±4.3	<0.001
IVS (mm)	10.5±1.3	11.2±1.4	0.001
LVPW (mm)	9.6±0.8	10±0.8	0.012
LVESD (mm)	27±2.3	28±3.3	<0.001
LVEDD (mm)	44.6±2.5	46.2±3.2	0.001

ACEi=angiotensin converting enzyme inhibitor; ARB=angiotensin receptor blocker; BMI=body mass index; CAD=coronary artery disease; DBP=diastolic blood pressure; HDL=High density lipoprotein; IVS=interventricular septum; LA=left atrium; LDL=low density lipoprotein; LVEDD=left ventricular end diastolic diameter; LVEF=left ventricular ejection fraction; LVESD=left ventricular end systolic diameter; LVPW=left ventricular posterior wall; MAC=mitral annular calcification; MetS=metabolic syndrome; OAD=oral antidiabetics; SBP=systolic blood pressure; TIA=transient ischemic attack

Smoking, coronary artery disease (CAD) and stroke/TIA rates were higher in patients with MAC than patients without MAC (*P*=0.016, *P*=0.026 and *P*=0.006, respectively). Hyperlipidemia and gender were similar between patients with and without MAC (for both parameters, *P*>0.05). MetS rates were higher in patients with MAC (*P*=0.001). Patients with MAC had higher CHA_2_DS_2_-VASc scores than patients without MAC (*P*<0.001). When, according to CHA_2_DS_2_-VASc score, patients were divided into two groups, with 0 and 1 scores considered as low risk, and ≥2 scores considered as high risk, patients with MAC had a higher rate of high risk category than patients without MAC (*P*<0.001). There was no statistically significant difference between two groups for cholesterol and glucose levels (for both parameters, *P*>0.05). LA diameter was significantly higher in patients with MAC than without MAC (37.4±4.3 *vs*. 34.7±4.8 mm, respectively; *P*<0.001). IVS thickness was significantly higher in patients with MAC than without MAC (11.2±1.4 *vs*. 10.5±1.3 mm, respectively; *P*=0.001). LVEDD was significantly higher in patients with MAC than without MAC (46.2±3.2 *vs*. 44.6±2 mm, respectively; *P*=0.001). LVESD was significantly higher in patients with MAC than without MAC (28±3.3 *vs*. 27±2.3 mm, respectively; *P*<0.001).

### Binary Logistic Regression for MAC

Correlation analysis indicated that MAC was positively correlated with CHA_2_DS_2_-VASc score (*P*<0.001, r=0.49), AF (*P*=0.008, r=0.208), LA diameter (*P*=0.001, r=0.267), age (*P*<0.001, r=0.347), history of hypertension (*P*<0.001, r=0.288), history of diabetes mellitus (*P*=0.001, r=0.261), presence of MetS (*P*=0.001, r=0.264), history of smoking (*P*=0.021, r=0.179), history of cerebrovascular accident/transient ischemic attack (CVA/TIA) (*P*=0.008, r=0.208), presence of AF (*P*=0.008, r=0.208), and negatively correlated with LVEF (*P*=0.021, r= -0.18) (Table 2).

**Table 2:** Correlation of mitral annular calcification with variables.

		CHA_2_DS_2_-VASc score	Age	Hypertension	Diabetes mellitus	LA diameter	LVEF	MetS	Smoking	CVA/TIA	AF
MAC	r	0.490	0.347	0.288	0.261	0.267	-0.180	0.264	0.179	0.208	0.208
	*P*	<0.001	<0.001	<0.001	0.001	0.001	0.021	0.001	0.021	0.008	0.008

AF=atrial fibrillation; CVA=cerebrovascular accident; LA=left atrium; LVEF=left ventricular ejection fraction; TIA=transient ischemic attack

**Fig. 1:**
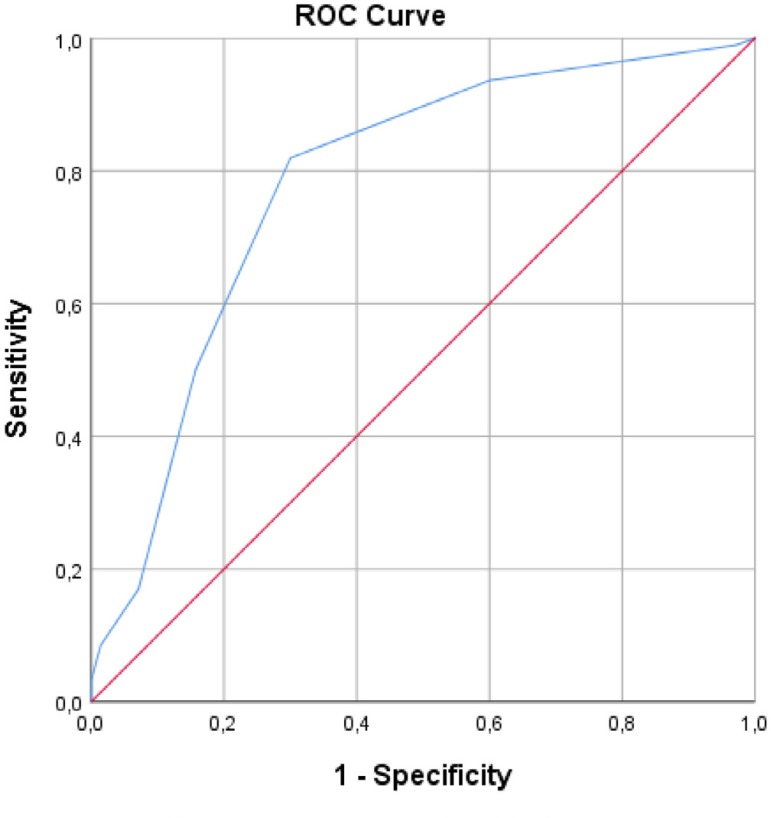
ROC curve with calculated area under the curve and optimal cut-off point for CHA2DS2-VASc score to identify the presence of MAC. C-statistic (area under the curve) — 95% conﬁdence interval (95% CI): CHA2DS2-VASc: (C-statistic: 0.78; 95% CI: 0.706-0.855, P<0.001). We calculated the cut-off point of 2.5 for CHA2DS2-VASc score to estimate the presence of MAC with a sensitivity of 81% and 70%.

Univariate analysis showed that high CHA_2_DS_2_-VASc score and group, presence of MetS, advanced age, history of hypertension, history of diabetes mellitus and history of smoking were significantly associated with a higher risk of MAC (Table 3). A multivariate binary logistic regression analysis was carried out including all the characteristics associated with MAC in the univariate analysis. This analysis showed that high risk according to the CHA_2_DS_2_-VASc score (OR: 5.0; 95% CI: 2.0-12.5, *P*<0.001), presence of MetS (OR: 1.002; 95% CI: 1.00-1.003, *P*=0.014) and history of smoking (OR: 2.09; 95% CI: 1.00-4.35, *P*=0.049) remained as independent factors for MAC. ROC curve analysis showed that CHA_2_DS_2_-VASc score (C-statistic: 0.78; 95% CI: 0.706-0.855, *P*<0.001) were significant predictors of MAC (Figure 1). We calculated that a cut-off point of 2.5 for CHA_2_DS_2_-VASc scores could estimate the presence of MAC with a sensitivity of 81% and 70%. When univariate analysis was carried out for CVA/TIA, presence of MAC increased 10-fold the CVA/TIA risk (OR: 10, 09; 95% CI: 1.28-79.62, *P*=0.028).

**Table 3:** Predictors of mitral annular calcification in univariate and multivariate logistic regression analysis.

	Unadjusted OR	CI 95%	*P*-value	Adjusted OR	CI 95%	*P*-value
Age, years	1.106	1.04-1.16	<0.001			
Hypertension	3.33	1.74-6.37	<0.001			
Diabetes mellitus	3.11	1.58-6.15	0.001			
History of smoking	2.16	1.11-4.21	0.023	2.09	1.00-4.35	0.049
MetS	2.98	1.57-5.6	0.001	2.44	1.20-4.96	0.014
CHA_2_DS_2_-VASc group (high risk)	7.1	3.0-17.0	<0.001	5.0	2.0-12.5	<0.001
CHA_2_DS_2_-VASc score	2.26	1.68-3.0	<0.001			

## DISCUSSION

The current study showed that higher CHA_2_DS_2_-VASc scores and presence of MetS were independently associated with the development of MAC; consequently, individuals at high risk according to CHA_2_DS_2_-VASc scores and presence of MetS should receive more attention to reduce unfavorable cardiovascular risk factors and the development of future cardiovascular events. The other finding of the study determined that the presence of MAC increased the risk of CVA/TIA and AF.

CHA_2_DS_2_-VASc score was associated with in-hospital and long-term adverse clinical outcomes, including mortality, in patients with stable CAD and acute coronary syndrome ^[14,15]^. To the best of our knowledge, there is no study investigating the relationship between CHA_2_DS_2_-VASc score, MetS and MAC, which has similar risk factors for atherosclerotic heart disease.

Since MAC is often associate with coronary risk factors, such as hypertension, diabetes and obesity^[2]^, it is possible that convergence of multiple risk factors could potentiate cardiovascular risk. This is exemplified by MetS. MAC is largely thought to be a manifestation of atherosclerotic coronary heart disease. This is mainly because the risk factors for MAC have been found to be similar to that of coronary artery disease, sharing a common pathogenesis for atherosclerosis.

MAC is associated with several cardiovascular disorders, including CAD, carotid and aortic atherosclerosis, heart failure, and stroke^[2,4,8]^. Previous studies have determined that age, hypertension, diabetes mellitus, and obesity, which are risk factors for atherosclerotic heart disease, are also risk factors for MAC^[2,16]^. In our study, we have found higher rates of diabetes mellitus and hypertension, which are a component of MetS and CHA_2_DS_2_-VASc score, in patients with MAC than in controls, and our results are consistent with previous studies of this aspect^[2,16]^. Bone morphogenic protein (BMP) 2, which is the key of osteogenic regulatory factor, is upregulated by hyperglycemia in vitro^[17,18]^. BMP2 showed to play a role in vascular calcification^[17]^ and has been detected in areas of valvular calcification^[18]^. Another theory that explained increased calcium in mitral annulus is the pro-oxidative status in diabetes mellitus. Oxidative stress (OS) is caused by increased production of reactive oxygen species. Increased oxidative products lead to cell death or to acceleration in ageing and age-related disease^[19]^. Besides, OS not only plays a role in the pathogenesis of MAC and may explain increased mortality and morbidity in patients with MAC. OS is also known to regulate different stages of thrombotic processes, including platelet activation. OS may trigger platelet hyperactivity, reducing nitric oxide bioavailability^[20]^. Additionally, OS have a role in the metabolic syndrome process^[21]^. Thus, increasing OS may play a role in increased mortality and morbidity in patients with MAC.

Studies have demonstrated that MetS was associated with increased rates of aortic stenosis progression and bioprosthetic valve deterioration^[22,23]^. In Multi-Ethnic Study of Atherosclerosis (MESA) cohort, Katz et al.^[24]^showed that MetS was associated with new and older aortic valve. In our study, we demonstrated that patients with MetS had higher MAC rates than patients without MetS. In the MESA^[5]^, investigators examined the association between MAC and AF in a racially and ethnically diverse population. They found that MAC was associated with an increased risk of AF (HR=1.9, 95% CI=1.5, 2.5). Hypertension and diabetes mellitus were more seen in patients with MAC than without MAC. Additionally, patients with MAC had left ventricular hypertrophy and left atrial enlargement more explicit than patients without MAC. Similarly, in the present study, we found that hypertension and diabetes mellitus were more observed in patients with MAC than without MAC. In addition, we have also found higher left atrial diameter, lower ejection fraction and higher incidence of left ventricular hypertrophy in patients with MAC than in controls. These findings may explain the association of MAC with adverse cardiovascular events, including stroke and increased mortality. Our other finding that AF and CVA/TIA were more seen in patients with MAC than without MAC, was consistent with left atrial dilatation and left ventricular hypertrophy. Our results are consistent with previous studies also in this respect^[3,5]^.

The global prevalence of MetS has increased due to the increased rates of obesity and sedentary lifestyle and has been associated with a higher risk of major adverse cardiac events in the general population^[25]^. Individuals with MetS should receive more attention to reduce unfavorable cardiovascular risk factors and the development of future cardiovascular events. Additionally, lifestyle changes and cardiovascular risk modification may reduce cardiac structural changes, such as left atrial dilatation, left ventricular hypertrophy and MAC formation.

## CONCLUSION

In conclusion, we showed that CHA_2_DS_2_-VASc score and presence of MetS rates were significantly higher in patients with MAC compared with controls. MAC was correlated with CHA_2_DS_2_-VASc score, presence of MetS, AF and left atrial diameter and negatively correlated with LVEF. The CHA_2_DS_2_-VASc score and presence of MetS were independently associated with presence of MAC. Presence of MetS and high CHA_2_DS_2_-VASc scores may indicate that patients with MAC have a higher risk of systemic thromboembolism.

**Table:** 

Authors' roles & responsibilities	
FA	Substantial contributions to the conception or design of the work; or the acquisition, analysis, or interpretation of data for the work; drafting the work or revising it critically for important intellectual content; final approval of the version to be published
SG	Substantial contributions to the conception or design of the work; or the acquisition, analysis, or interpretation of data for the work; final approval of the version to be published
FK	Substantial contributions to the conception or design of the work; or the acquisition, analysis, or interpretation of data for the work; drafting the work or revising it critically for important intellectual content; final approval of the version to be published
MSK	Agreement to be accountable for all aspects of the work in ensuring that questions related to the accuracy or integrity of any part of the work are appropriately investigated and resolved
AB	Substantial contributions to the conception or design of the work; or the acquisition, analysis, or interpretation of data for the work; drafting the work or revising it critically for important intellectual content; final approval of the version to be published
HAB	Agreement to be accountable for all aspects of the work in ensuring that questions related to the accuracy or integrity of any part of the work are appropriately investigated and resolved
DU	Drafting the work or revising it critically for important intellectual content
EV	Substantial contributions to the conception or design of the work; or the acquisition, analysis, or interpretation of data for the work; drafting the work or revising it critically for important intellectual content; final approval of the version to be published
